# Tau Ser208 phosphorylation promotes aggregation and reveals neuropathologic diversity in Alzheimer’s disease and other tauopathies

**DOI:** 10.1186/s40478-020-00967-w

**Published:** 2020-06-22

**Authors:** Yuxing Xia, Stefan Prokop, Kimberly-Marie M. Gorion, Justin D. Kim, Zachary A. Sorrentino, Brach M. Bell, Alyssa N. Manaois, Paramita Chakrabarty, Peter Davies, Benoit I. Giasson

**Affiliations:** 1 Department of Neuroscience, College of Medicine, University of Florida, 1275 Center Drive, Gainesville, Florida, 32610 USA; 2 Center for Translational Research in Neurodegenerative Disease, College of Medicine, University of Florida, 1275 Center Drive, Gainesville, Florida, 32610 USA; 3 McKnight Brain Institute, College of Medicine, University of Florida, 1275 Center Drive, Gainesville, Florida, 32610 USA; 4 Department of Pathology, College of Medicine, University of Florida, Gainesville, FL 32610 USA; 5 Norman Fixel Institute for Neurological Diseases, College of Medicine, University of Florida, Gainesville, FL 32610 USA; 6 Litwin-Zucker Center for the Study of Alzheimer’s Disease, The Feinstein Institute for Medical Research, Northwell Health, Manhasset, New York, USA

**Keywords:** Alzheimer’s disease, tau, Neurodegeneration, Neurofibrillary tangle, Microtubule-associated protein tau (MAPT), Protein aggregation, AT8, CP13, Microtubule binding, Prion-like seeding, Tauopathy

## Abstract

Tau protein abnormally aggregates in tauopathies, a diverse group of neurologic diseases that includes Alzheimer’s disease (AD). In early stages of disease, tau becomes hyperphosphorylated and mislocalized, which can contribute to its aggregation and toxicity. We demonstrate that tau phosphorylation at Ser208 (pSer208) promotes microtubule dysfunction and tau aggregation in cultured cells. Comparative assessment of the epitopes recognized by antibodies AT8, CP13, and 7F2 demonstrates that CP13 and 7F2 are specific for tau phosphorylation at Ser202 and Thr205, respectively, independently of the phosphorylation state of adjacent phosphorylation sites. Supporting the involvement of pSer208 in tau pathology, a novel monoclonal antibody 3G12 specific for tau phosphorylation at Ser208 revealed strong reactivity of tau inclusions in the brains of PS19 and rTg4510 transgenic mouse models of tauopathy. 3G12 also labelled neurofibrillary tangles in brains of patients with AD but revealed differential staining compared to CP13 and 7F2 for other types of tau pathologies such as in neuropil threads and neuritic plaques in AD, tufted astrocytes in progressive supranuclear palsy and astrocytic plaques in corticobasal degeneration. These results support the hypothesis that tau phosphorylation at Ser208 strongly contributes to unique types of tau aggregation and may be a reliable marker for the presence of mature neurofibrillary tangles.

## Introduction

Alzheimer’s disease (AD) is the most common form of age-related dementia and affects over 25 million people worldwide [66]. AD is pathologically defined by the presence of two major types of pathologic brain inclusions: 1) extracellular amyloid-β (Aβ) deposits in the form of plaques and cerebral amyloid angiopathy and 2) intracellular aggregates of tau protein that comprise neurofibrillary tangles (NFT) and neuropil threads [11, 40]. The amyloid cascade hypothesis suggests that the accumulation and deposition of Aβ is the primary cause of AD [70]. However as the density of tau inclusions strongly correlates with cognitive decline [63], the “tau hypothesis” proposes that pathogenic tau is the main toxic factor that drives neurodegeneration in AD and other related diseases [43]. Mutations in the microtubule-associated protein tau (MAPT) gene cause different familial forms of frontotemporal lobar degeneration (FTLD) including Pick’s disease (PiD), progressive supranuclear palsy (PSP), corticobasal degeneration (CBD), and globular glial tauopathy (GGT) [23, 39, 80].

Tau is a microtubule-associated protein that is highly expressed in the distal axons of neurons in the central nervous system [9, 80]. Physiologically, tau binds directly to microtubules (MTs) and is important for regulating MT assembly, dynamics, and stability, which are all important for normal axonal transport of vesicles and other molecules [42, 44, 83]. Tau protein can be alternatively spliced into six major isoforms found in the brains of humans and rodents [30, 31]. Variations in insertion of two N-terminal inserts of 29 amino acids generate 0 N, 1 N and 2 N isoforms. The presence or absence of exon 10 can lead to either three or four MT binding repeats to generate either 3R or 4R isoforms.

In AD, all six major tau isoforms aggregate to form pathological inclusions in a hyperphosphorylated state [29, 80]. Over 45 out of 85 total potential phosphorylation sites have been identified in AD brains by mass spectrometry and other methods [34–37, 60]. This aberrant hyperphosphorylation can cause tau to dissociate from MTs and decrease its ability to assemble and regulate MTs [22, 47, 49, 71]. Hyperphosphorylated tau may also be more prone to be mislocalized and to promote tau aggregation [2, 12, 32, 75]. Phosphorylation-specific tau antibodies such as AT8 are widely used to survey the distribution of tau pathology in AD brains, which follows a stereotypical pattern of progression as described in Braak staging – with NFT appearing to start in the entorhinal cortex and extending into the hippocampus and more distant cortical regions in late stage disease [10, 11].

Although the AT8 antibody is very useful diagnostic tool, the exact epitope recognized by this antibody has not been completely resolved, since AT8 was originally created by immunizing mice with paired helical filaments of tau (PHF-tau) purified from AD brains [58]. Early characterization of the AT8 epitope indicated that it requires phosphorylation at Ser202 (pSer202) and Thr205 (pThr205) [8, 28]; however, AT8 also has some reactivity to tau phosphorylation at Ser199 [8]. Recent epitope mapping has revealed that tau pSer208 may also be a part of the classic AT8 epitope and the addition of pSer208 to pSer202/pThr205 can enhance AT8 binding [53, 65]. Phosphorylation of Ser208 has separately been found in mass spectrometry analysis of PHF-tau purified from AD brains [34, 60]. In the cerebrospinal fluid of AD patients, pSer208 levels were three times more elevated compared to healthy controls [6]. Recent in vitro studies have indicated that triple phosphorylation of Ser202/Thr205/Ser208 promotes tau aggregation [19]; however, this has not been confirmed in cell culture, mouse models, or and human postmortem studies.

In this study, we modeled tau phosphorylation at Ser208 and nearby phosphorylation sites with phosphomimetics to determine its role in tau aggregation and MT binding in cultured cells. To confirm the in vivo relevance of pSer208, we also created a novel monoclonal antibody 3G12 specific for pSer208 and demonstrated its strong reactivity for tau inclusions in transgenic mouse models of tauopathies and in postmortem brain samples of patients with AD and other tauopathies.

## Materials and methods

### K18 tau protein purification

The K18 tau fragment consists of the MT binding repeats of the 2N4R human tau protein isoform, which includes amino acid residues Q244 to E372. An additional N-terminal methionine was added to K18 protein and expressed under the bacterial plasmid pRK172 in BL21 (DE3)/RIL *Escherichia coli* (Agilent Technologies, Santa Clara, CA). Recombinant K18 tau protein was purified as previous described [17, 72, 84]. Protein concentration was determined using a bicinchoninic acid assay (Thermo Fisher Scientific, Waltham, MA) and albumin for the standard curve.

### Fibrillization of tau K18 seeds

Purified K18 tau protein was dissolved in PBS at a concentration of 1 mg/mL and 50 μM of heparin and was placed in a shaking incubator at 1050 RPM and 37 °C for at least 2 days. As previously described, the presence of polymerized amyloidogenic K18 fibrils structure was confirmed by K114 or thioflavin T assays [17]. To remove heparin, K18 tau fibrils were centrifuged at 100,000 g for 30 min and re-dissolved in PBS followed by water bath sonication for 60 min resulting in shorter tau fibrils [72, 81, 84].

### Mammalian tau expression plasmids and site-directed mutagenesis

The 2N4R human tau isoform cDNA was cloned into the pcDNA3.1 mammalian expression vector. Phosphomimetic mutations were introduced by QuikChange site-directed mutagenesis (Agilent Technologies, Santa Clara, CA) with customized oligonucleotides. The sequence of all constructs with the entire tau sequence was verified by Sanger sequencing performed by Genewiz (South Plainfield, NJ).

### HEK293T cultured cells and calcium phosphate transfection

HEK293T cells were maintained at 37 °C and 5% CO_2_ in Dulbecco’s modified Eagle’s media and 10% fetal bovine serum (FBS) supplemented with antibiotics (100 units/ml penicillin, 100 μg/ml streptomycin). Calcium phosphate precipitation was used to transfect HEK293T cells with various plasmid constructs. Cells were split into 12-well plates at 20–40% confluency. For each well, 1.5 μg of DNA was mixed with 18.75 μL of 0.25 M CaCl_2_. This mixture was added to an equivalent of 2X BES buffer (50 mM BES, 280 mM NaCl, 1.5 mM Na_2_HPO_4_, pH 6.96) and incubated at room temperature for 15–20 min. The final solution was placed dropwise to each well. For tau seeding experiments, 1 μM of purified K18 tau fibrils was added an hour post-transfection [72, 84]. 16 h after transfection, cells were washed with PBS and placed in 3% FBS until they were harvested at 48 h after the media change.

### Cellular tau aggregation assay

HEK293T cells were harvested in 200 μL of Triton Lysis Buffer (25 mM Tris-HCl, pH 7.5, 150 mM NaCl, 1 mM EDTA, 1% Triton X-100, 20 mM NaF) with a mix of different protease inhibitors as previously described [72, 84]. Cell lysates were centrifuged at 100,000 g and 4 °C for 30 min to separate into a soluble and insoluble fraction. The insoluble fraction was washed in additional buffer and centrifuged again at 100,000 g and 4 °C for 30 min. The pellets were resuspended in Triton Lysis Buffer. Both fractions were boiled for 10 min after adding SDS- sample buffer (10 mM Tris, pH 6.8, 1 mM EDTA, 40 mM DTT, 0.005% bromophenol blue, 0.0025% pyronin yellow, 1% SDS, 10% sucrose). The insoluble fraction was sonicated and boiled again for 10 min to completely dissolve the pellet.

### Cellular MT binding assay

HEK293T cells were lysed in 200 μL of PEM buffer (80 mM PIPES, pH 6.8, 1 mM EGTA, 1 mM MgCl_2_) supplemented with 0.1% Triton X-100, 2 mM GTP, 20 μM paclitaxel, and protease inhibitors as previously described [77, 84]. Cell lysates were incubated at 37 °C for 30 min and centrifuged at 100,000 g for 30 min to isolate MTs. Supernatants were transferred to a new tube and the pellet (MT fraction with bound proteins) were resuspended in PEM buffer. The pellet fraction was homogenized and SDS-sample buffer was added to both fractions. Equivalent amounts of supernatant and pellet were loaded on SDS polyacrylamide gels for Western Blot analysis. Percentage of MT-bound tau was calculated with pellet / (supernatant + pellet) * 100.

### Enzyme-linked Immunosorbent assay (ELISA)

96-well ELISA plates (Corning Life Sciences, Corning, NY) were coated with 100 ng in 100 μL PBS per well of each peptide (see Table 1). All wells were washed with PBS four times and blocked with PBS with 5% FBS. Primary antibodies were added to blocking solution and incubated for 1 h. After PBS washes, plates were incubated with horseradish peroxidase-conjugated goat anti-mouse antibody (Vector Labs Inc., Burlingame, CA) in blocking solution for an hour. Plates were washed with PBS and 3,3′,5,5′-tetramethylbenzidine (TMB substrate, Thermo Fisher Scientific, Waltham, MA) was added to each well. The reactions were stopped with 0.2 M HCl and the optical density was measured at 450 nm with a plate reader. All ELISA experiments were performed in quadruplicates.

**Table 1:** Table of synthetic peptides used for ELISAs

Peptide Name	Peptide Sequence
pSer199, pSer202, pSer205	^193^DRSGYS-**pS**-PG-**pS**-PG-**pT**-PGSRSR^211^-Cys
pSer199	^193^DRSGYS-**pS**-PGSPGTPGSRSR^211^-Cys
pSer202	^193^DRSGYSSPG-**pS**-PGTPGSRSR^211^-Cys
pThr205	^193^DRSGYSSPGSPG-**pT**-PGSRSR^211^-Cys
pSer208	Cys-^202^SPGTPG-**pS**-RSRTP^213^
pThr205/pSer208	Cys-^202^SPG-**pT**-PG-**pS**-RSRTP^213^
Ser208	Cys-^202^SPGTPGSRSRTP^213^

### Generation of monoclonal antibody to tau phosphorylated at Ser208

BALC/c mice (Jackson Laboratory, Bar Harbor, ME) were immunized with a synthetic peptide Cys-^202^SPGTPGpSRSRTP^213^ (synthesized and purified by GenScript USA Inc., Piscataway, NJ), which corresponds to tau phosphorylated at Ser208, conjugated to KLH as previously described [16, 73, 74]. Hybridoma clones were screened for their specificity by ELISA [16, 73]. Monoclonal antibody 3G12 was found to be specific for tau phosphorylated at Ser208 by ELISA and was useful for immunohistochemistry and western blotting. All of the synthetic peptides used are listed in Table 2 and were synthesized and purified by GenScript USA Inc. (Piscataway, NJ).

**Table 2:** List of Tau Antibodies

Antibody	Specificity	Peptide/Protein Used for Immunization
AT8	Combinations of pSer202, pThr205, pSer208	PHF-tau from AD brain [8, 58]
CP13	pSer202	PHF-tau from AD brain^a^
7F2	pThr205	^193^DRSGYS-**pS**-PG-**pS**-PG-**pT**-PGSRSR^211^-Cys [73]
3G12	pSer208	Cys-^202^SPGTPG-**pS**-RSRTP^213^
2D1	Phosphorylation independent	^193^DRSGYS-**pS**-PG-**pS**-PG-**pT**-PGSRSR^211^-Cys [73]
3026	Total tau	recombinant full-length 0N/3R human tau [73]

^a^Personal communication with Dr. Peter Davies

### Western blot and semi-quantitative analysis

Protein samples were loaded on 10% polyacrylamide gels for SDS-PAGE and electrophoretically transferred to nitrocellulose membranes. Membranes were blocked in 5% milk in TBS for 1 h at room temperature and incubated in primary antibodies overnight at 4 °C at 1:1000 dilutions for 3026 total tau antibody [73], β-tubulin antibody (Clone TUB 2.1 from Sigma-Aldrich, St. Louis, MO), and tau antibody 3G12 specific for pSer208. After TBS washes, the membranes were added to anti-rabbit or anti-mouse secondary antibodies conjugated to horseradish peroxidase (Jackson ImmunoResearch, West Grove, PA) for 1 h. After TBS washes, the membranes were reacted with Western Lightning Plus ECL reagents (PerkinElmer Life Sciences, Waltham, MA) and the signal was captured by chemiluminescence imaging (PXi, Syngene, Frederick, MD). The specific signals in each lane were quantified based on densitometric analysis with ImageJ software. Statistical tests were calculated on GraphPad Prism for one-way or two-way analysis of variance (ANOVA) with post hoc analysis by Dunnett’s test for group comparison.

### Generation of Triton X-100 insoluble fractionations of mouse brain tissue

Brain samples from non-transgenic (nTG), tau knockout (KO) [18] and rTg4510 transgenic tau [67, 69] mice were lysed in TBS buffer (50 mM Tris base, 274 mM NaCl, 5 mM KCl, pH  8.0) supplemented with 1% Triton X-100 and protease and phosphatase inhibitors and probed sonicated until the solution is homogenous. The brain lysates were centrifuged at 100,000 g for 30 min at 4 °C. The Triton-insoluble pellets fractions were resuspended in the same buffer and SDS-sample buffer was added with heated at 100 °C for 10 min before loading for SDS-PAGE.

### Immunohistochemistry of human and mouse brain tissue

Formalin-fixed brain samples of patients with AD, PSP, and CBD were provided by the University of Florida Neuromedicine Human Brain and Tissue Bank (UF HBTB) following institutional regulations. See Table 3 for details on human cases used for this study. Ethanol-fixed and formalin-fixed brain samples were obtained from PS19 transgenic mice that overexpress 1N4R human tau isoform with the P301S mutation [85] and rTg4510 transgenic mice that overexpress 0N4R human tau isoform with the P301L mutation, respectively [67, 69]. Paraffin-embedded tissue on slides were rehydrated in xylene and series of ethanol solutions (100, 90, and 70%). For standard heat antigen retrieval, slides were placed in a steam bath for 30 min to an hour in water supplemented with 0.05% Tween-20. Endogenous peroxidase was quenched by submerging slides in PBS solutions with 1.5% hydrogen peroxide and 0.005% Triton-X-100. After washing, slides were blocked in 2% FBS/0.1 M Tris, pH 7.6 and were incubated in primary antibody overnight at 4 °C. Primary antibody dilutions were 1:500 for AT8 antibody (Thermo Fisher Scientific, Waltham, MA), 1:1000 for 7F2 antibody [73], 1:250 for CP13 antibody [82] (Dr. Peter Davies), and 1:1000 for antibody 3G12 against Tau phosphorylated Ser208. After washes with 0.1 M Tris, pH 7.6, slides were sequentially incubated with biotinylated anti-mouse secondary antibody (Vector Laboratories, Burlingame, CA) for 1 h and streptavidin-conjugated HRP (Vectastain ABC kit from Vector Laboratories, Burlingame, CA) for 1 h. All slides were developed in 3, 3′-diaminobenzidine (DAB kit; KPL, Gaithersburg, MD) and counterstained with Mayer’s hematoxylin (Sigma Aldrich, St. Louis, MO). Slides were dehydrated in ethanol solutions (70, 90, and 100%) and xylene before they were covered with Cytoseal (Thermo Scientific, Waltham, MA).

**Table 3:** Demographic data of clinical cases, diagnoses and pathologic findings

	Age	Gender	Primary Diagnosis	Secondary Diagnosis	Thal Phase (A score)	Braak stage (B score)	CERAD (C score)
AD	78	female	ADNC, high	CAA	4 (A3)	VI (B3)	frequent (C3)
AD	64	female	ADNC, high	CAA	4 (A3)	VI (B3)	frequent (C3)
AD	77	male	ADNC, high	ARTAG; CAA	4 (A3)	VI (B3)	moderate (C2)
AD	64	male	ADNC, high	CAA	5 (A3)	VI (B3)	frequent (C3)
AD	68	male	LBD (neocortical)	ADNC, intermediate	3 (A2)	V (B3)	frequent (C3)
DLB	81	female	ADNC, high	LBD (neocortical); CAA	4 (A3)	VI (B3)	frequent (C3)
DLB	68	female	LBD (neocortical)	ADNC, high; CAA; LATE stage1	5 (A3)	V (B3)	frequent (C3)
DLB	83	male	ADNC, intermediate	LBD (neocortical); CAA; LATE stage 2	3 (A2)	V (B3)	frequent (C3)
PSP	63	female	FTLD-tau (PSP)		0 (A0)	0 (B0)	none (C0)
PSP	69	female	FTLD-tau (PSP)	ADNC, low	3 (A2)	III (B2)	none (C0)
PSP	72	female	FTLD-tau (PSP)	PART (Braak II)	0 (A0)	II (B1)	none (C0)
PSP	78	male	FTLD-tau (PSP)		0 (A0)	0 (B0)	none (C0)
PSP	77	male	FTLD-tau (PSP)	ADNC, low; CAA	2 (A1)	II (B1)	none (C0)
CBD	73	female	FTLD-tau (CBD)	ADNC, low; CAA	1 (A1)	II (B1)	none (C0)
CBD	70	male	FTLD-tau (CBD)	ADNC, low; CAA	3 (A2)	II (B1)	none (C0)

Primary neuropathologic diagnoses were based on current guidelines for Alzheimer’s disease neuropathologic change (ADNC) [59], dementia with Lewy Bodies (DLB) [55], and frontotemporal lobar dementia-tau (FTLD-tau) pathology [52], including progressive supranuclear palsy (PSP), and corticobasal degeneration (CBD) [20]. Additionally, secondary neuropathologic changes were determined following guidelines for cerebral amyloid angiopathy (CAA) [13], Aging-related tau astrogliopathy (ARTAG) [48], and limbic-predominant age-related TDP-43 encephalopathy (LATE) [64]

### Semi-quantitative analysis of pathological counts

For AD and dementia with Lewy body (DLB) cases (details in Table 3), three different raters (YX, SP and ZAS) scored NFT, neuritic plaques, and neuropil threads in the hippocampus and surrounding regions for phosphorylation-specific antibodies AT8 and 3G12. Pathologic hallmarks were counted in randomly selected 20X fields in CA4, CA3, CA2, CA1, subiculum, entorhinal cortex and adjacent inferior temporal cortex accounting for seven total regions scored per case. The number of NFT and tau-positive neurons were counted per 20X field, and the percent of mature tangles were calculated as a ratio of mature NFT to tau-positive neurons. Similarly, neuritic plaques were counted in the same randomly selected 20x fields. Neuropil threads were scored on a graded scale from 1 to 3 based on relative density. Between the three different raters, 168 different data points were calculated for statistical analysis of each type of brain pathology.

Using IBM SPSS Statistics program (IBM, Armonk, New York), interrater reliability was assessed by calculating the intraclass correlation coefficient (ICC) with a two-way mixed model for consistency between three different raters [33]. For counting of NFT, the intraclass correlation coefficient was 0.822 with a 95% confidence interval from 0.757 to 0.873. For counting of neuritic plaques, the intraclass correlation coefficient was 0.696 with a 95% confidence interval from 0.584 to 0.782. For scoring of neuropil threads, the intraclass correlation coefficient was 0.534 with a 95% confidence interval from 0.362 to 0.666. Based on these results, counting of NFT and neuritic plaques had good reliability for consistency between the three raters. Scoring of neuropil threads was moderately reliable between different raters.

## Results

### Triple tau phosphomimetic S202E/T205E/S208E promotes aggregation of wild type (WT) tau in cultured cells

**Fig. 1:**
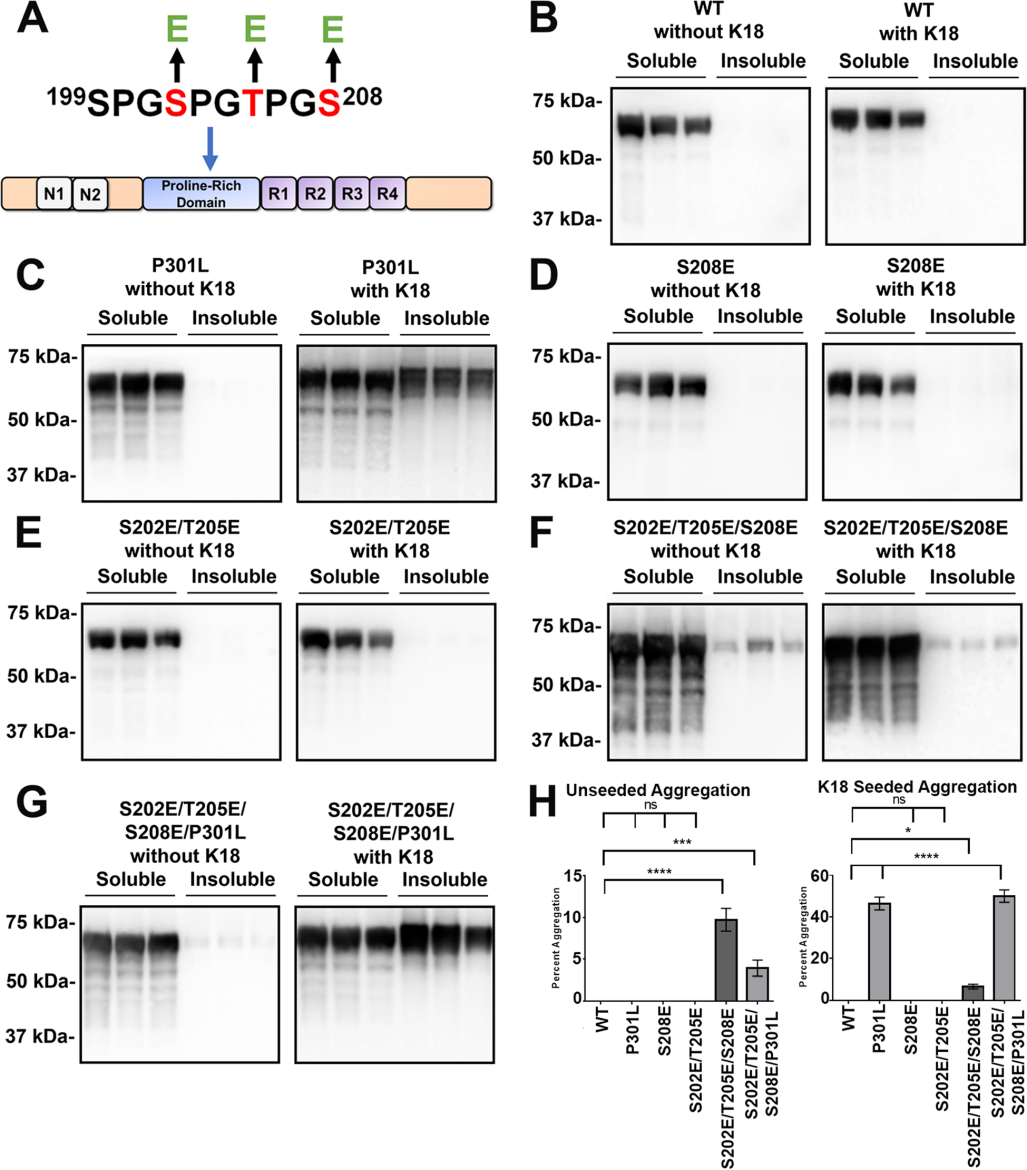
Triple phosphomimetic S202E/T205E/S208E promotes aggregation of WT tau. **a** Schematic of 2N4R human tau protein depicting the major domains and the expanded 199–208 amino acid region within the proline-rich domain. Ser202, Thr205, and Ser208 were mutated to Glu residues to model site-specific tau phosphorylation. **b** HEK293T cells were transfected to express 2N4R human WT tau (**b**), P301L tau mutant (**c**), or the indicated tau phosphomimetics (**d**-**g**) and were biochemically assessed for tau aggregation with or without the addition of K18 seeds as described in “Materials and Methods.” S = supernatant fraction, P = pellet fractions. Immunoblots were probed with total tau antibody 3026. **c** As a positive control for seeding with K18 seeds the tau P301L mutant was used. Similar aggregation and seeding studies were performed with the single phosphomimetic S208E (**d**), the double phosphomimetic S202E/T205E (**e**), and the triple phosphomimetic S202E/T205E/S208E (**f**). **g** Triple phosphomimetic S202E/T205E/S208E in the context of the P301L mutation was also assessed. The relative molecular masses of protein markers are indicated on the left. **h** Quantification of percent tau aggregation was determined as described in “Materials and Methods.” One-way ANOVA with Dunnett’s Test was performed with *N* = 6 for WT tau and *N* = 3 for each tau mutant. **** is *p* < 0.0001, *** is *p* < 0.001, * is *p* < 0.05 and ns = not statistically significant. Error bars show standard error of the mean

A previous in vitro study indicated that the combined phosphorylation of Ser202, Thr205, and Ser208 residues promotes the polymerization of tau into filaments [19]. To assess if this phosphorylation pattern could also promote aggregation in a cell model, site-specific tau phosphomimetics of Ser or Thr to Glu substitutions were created at these sites (Fig. 1a). Tau aggregation was assessed in the absence and presence of exogenous K18 tau amyloid seeds. Tau mutant P301L is prone to K18 seeded aggregation and was used as a positive control [25, 72, 84]. All types of tau including WT, P301L, and phosphomimetics were expressed at comparable levels (Supplemental Figure 1).

As previously shown [72, 84], WT tau does not significantly aggregate with or without K18 induced seeding (Fig. 1b, h), while P301L tau robustly aggregate in the presence of seeds (Fig. 1c, h). Single tau phosphomimetic S208E and double tau phosphomimetic S202E/T205E did not significantly aggregate with or without seeding (Fig. 1d, e, h). In contrast, the triple tau phosphomimetic S202E/T205E/S208E showed some aggregation without seeding but this was not enhanced by the addition exogenous K18 tau seeds (Fig. 1f, h). Combining the triple tau phosphomimetic S202E/T205E/S208E with the P301L mutation did not have an additive effect on tau aggregation more than P301L by itself (Fig. 1g).

### S208E tau phosphomimetic modulates MT binding

**Fig. 2:**
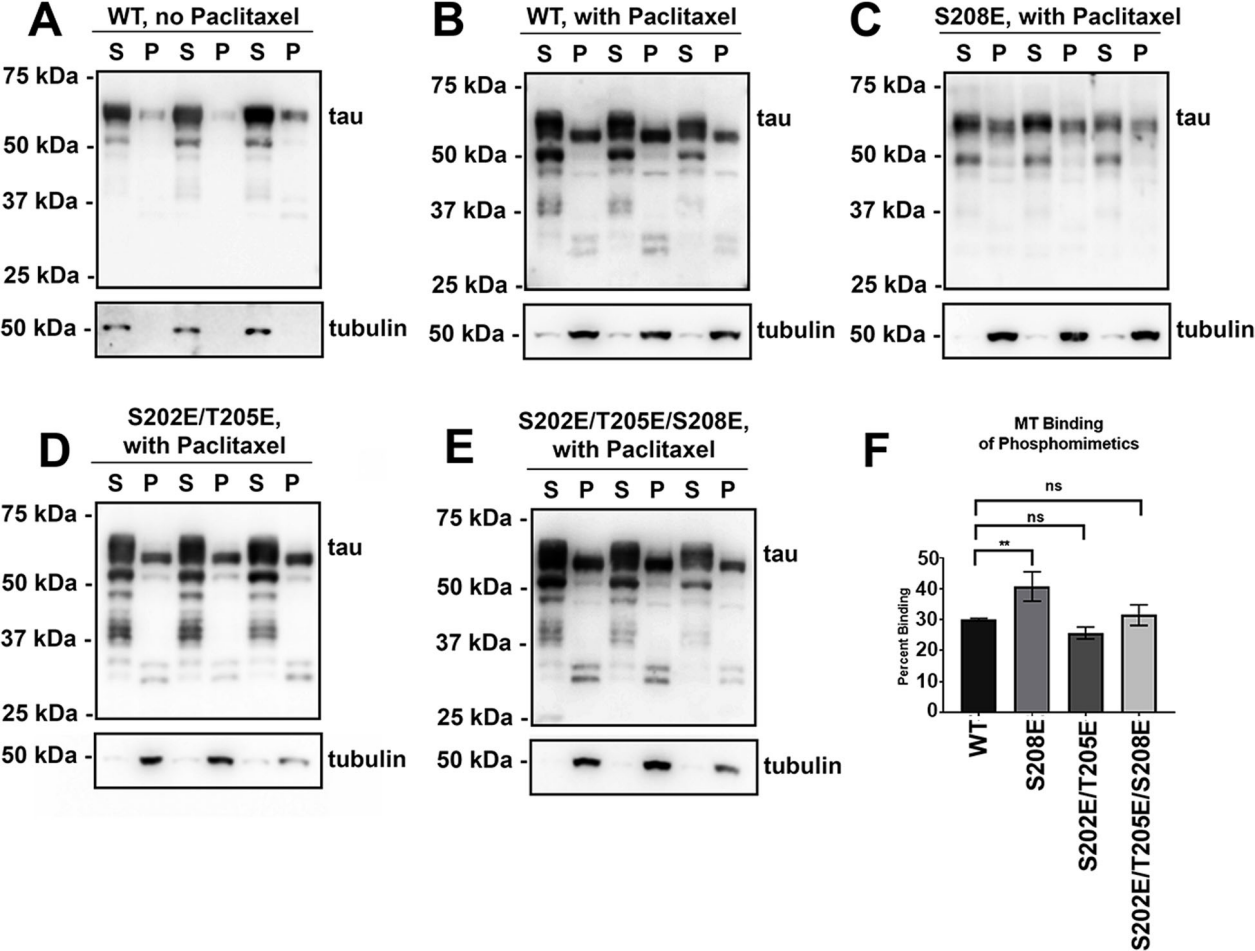
Tau phosphomimetic S208E increased MT binding compared to WT tau and other tau phosphomimetics. **a**, **b** Cell-based MT binding assays were performed on HEK293T cells transfected to express 2N4R WT human tau without (**a**) or with (**b**) Paclitaxel added as described in “Material and Methods”. The same assay with Paclitaxel added as performed for cells expressing 2N4R human tau with the (**c**) S208E, (**d**) S202E/T205E or (**e**) S202E/T205E/S208E phosphomimetics. Immunoblots were probed with antibody specific for β-tubulin (clone TUB 2.1) to confirm tubulin polymerization or with 3026, a polyclonal antibody against total tau. S = supernatant fraction; P = pellet fractions. The relative molecular masses of protein markers are indicated on the left. **f** Quantification of percent tau associated with MTs. One-way ANOVA with Dunnett’s Test was performed with *N* = 3 for WT tau and *N* = 3 for each of these tau mutants. ** = *p* < 0.01, ns = not statistically significant. Error bars show standard error of the mean

Prior in vitro studies have suggested that phosphorylation of Ser202 and Thr205 can decrease tau’s ability to promote MT polymerization [78], but the effects of phosphorylated Ser208 have not been investigated. Therefore, MT binding of different phosphomimetics was assessed using a cell-based assay [1, 38, 77, 84]. In this assay, paclitaxel is used to stabilize MT and proteins that bind to MT such as tau can be pulled down by high-speed centrifugation. When paclitaxel is added to cell lysates, stable MT are formed and are present in the pellet fraction. As a MT binding protein, tau is also increased in the pellet fraction. In WT control samples, where the same cell lysates were not treated with paclitaxel, most of the unpolymerized tubulin is present in the soluble fraction where also the majority of tau is found, with only ~ 10% in the pellet fraction (Fig. 2a). In the presence of paclitaxel, tubulin polymerized as MT is predominantly present in the pellet fraction and WT 2N4R tau binds MTs at about 30% (Fig. 2b, f). In contrast, S208E tau shows increased MT binding at ~ 41% compared to WT tau (Fig. 2c, f). Surprisingly, tau phosphomimetics S202E/T205E and S202E/T205E/S208E did not show significant changes in MT binding relative to WT tau (Fig. 2d, e, f).

### Generation and characterization of novel monoclonal antibody 3G12 specific for tau phosphorylated at Ser208

**Fig. 3:**
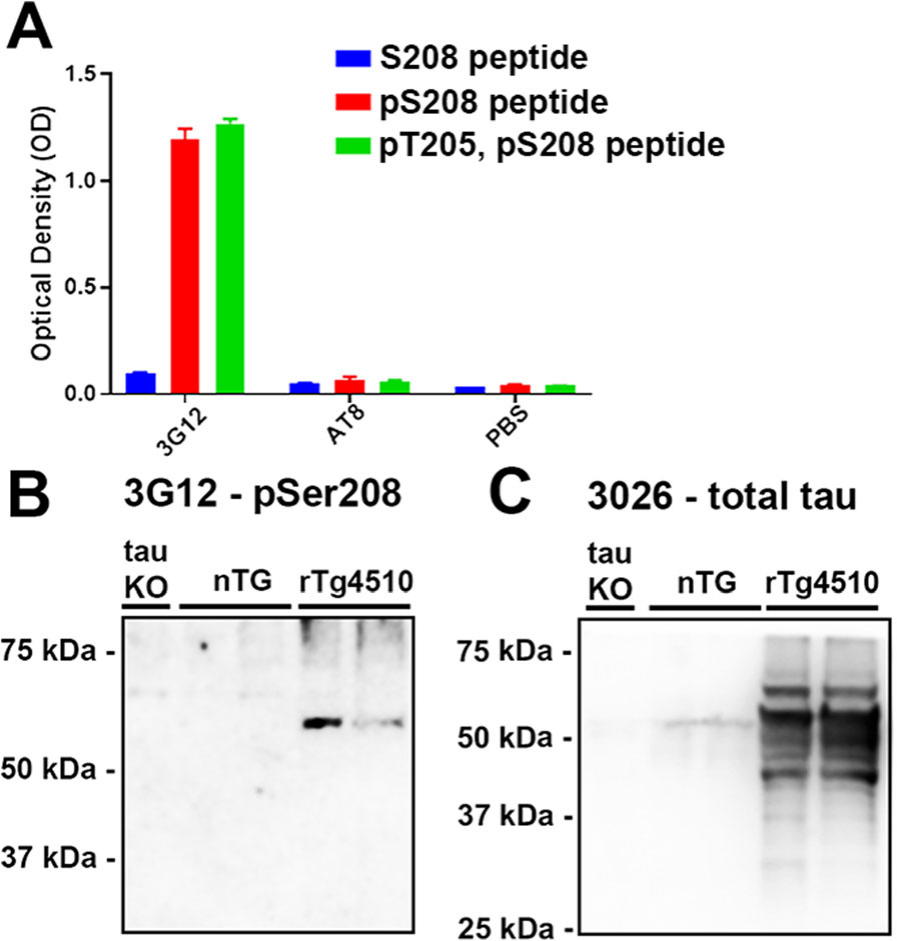
Characterization of the specificity of a new monoclonal antibody 3G12 against pSer208. **a** ELISA was performed to assess the specificity of 3G12 monoclonal antibody compared to AT8 and PBS controls. Peptides used in ELISA are shown in Table 1. **b** Brain Triton X-100 insoluble fractions from tau knockout mice (tau KO), nontransgenic mice (nTG), and 6 month old rTg4510 tau transgenic mice were loaded on 10% polyacrylamide gels for immunoblotting analysis. Membranes were probed with (**b**) 3G12 monoclonal antibody or (**c**) rabbit polyclonal antibody 3026. The relative molecular masses of protein markers are indicated on the left

To study tau phosphorylation of Ser208 (pSer208) in human tauopathies, a new monoclonal antibody specific for pSer208 was generated by immunizing mice as described in Material and Methods using the synthetic pSer208 peptide (Table 2). By ELISA, monoclonal antibody 3G12 strongly reacted with the pSer208 peptide but not with the corresponding non-phosphorylated peptide (Fig. 3a). Since the Thr205 residue is in proximity with Ser208, we tested if phosphorylation of Thr205 could interfere with 3G12 binding. 3G12 bound to the dual phosphorylated pThr205/pSer208 peptide with a similar efficiency as the phosphorylated Ser208 peptide, suggesting Thr205 phosphorylation does not block 3G12 antibody from binding to the pSer208 epitope (Fig. 3a). For comparison, the AT8 antibody did not react with any of these peptides (Fig. 3a).

To test the specificity of antibody 3G12 and the presence of tau Ser208 phosphorylation within aggregated tau in disease models, immunoblot analysis was performed using the Triton-insoluble fraction of brain lysates of tau KO, nTG mice, and rTg4510 transgenic mice that overexpress 0N4R isoform of P301L tau mutation [67, 69]. 3G12 antibody detected aggregated tau in rTg4510 transgenic mice and shows no major nonspecific bands in nTG mice or tau KO mice (Fig. 3b). A total tau antibody 3026 was used to confirm the presence of aggregated human tau in rTg4510 transgenic mice (Fig. 2c).

**Fig. 4:**
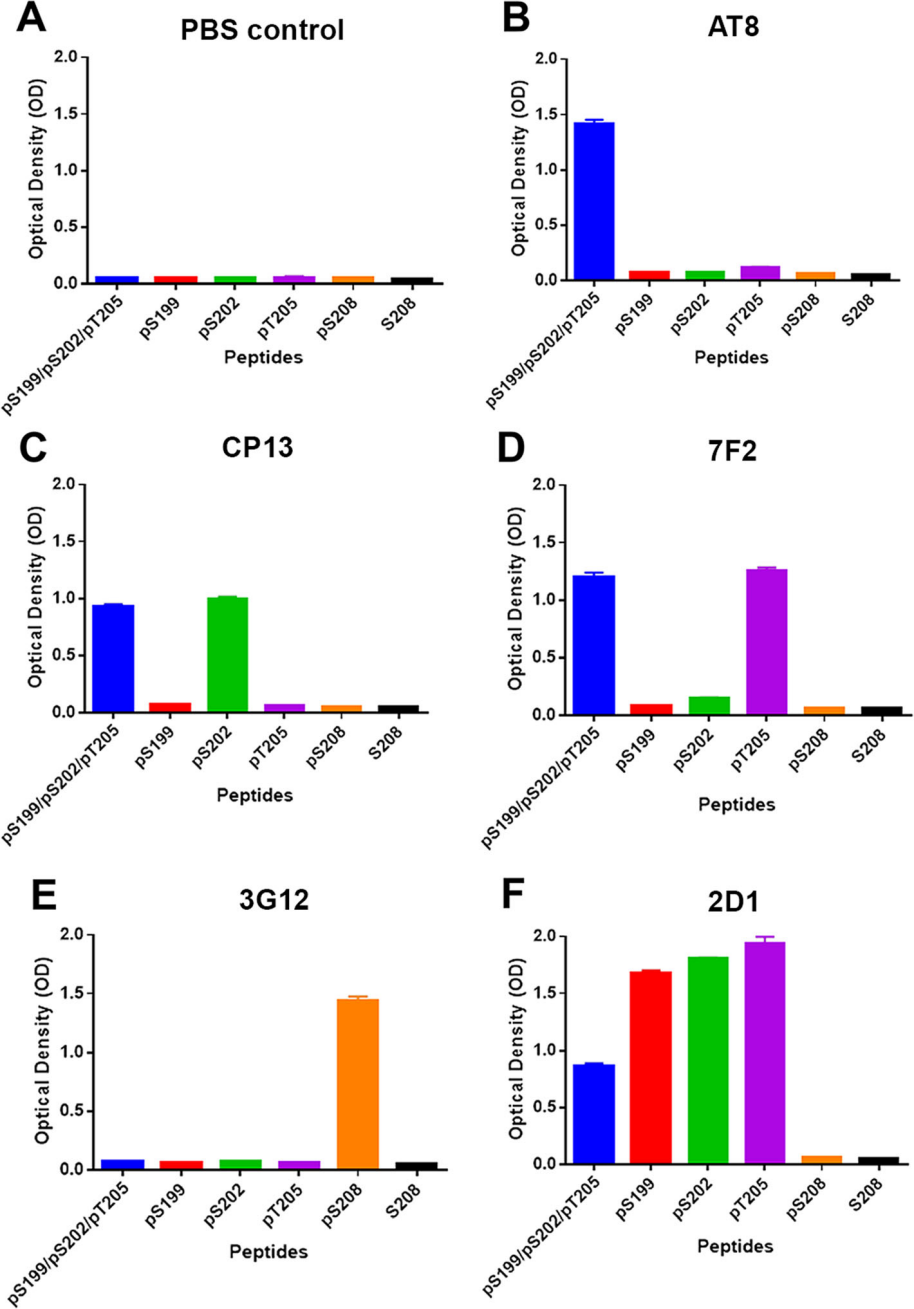
ELISA analysis of phospho-tau specific antibodies with different peptides near the AT8 Epitope. ELISA was used to assess the specificity of tau monoclonal antibodies AT8, CP13, 7F2, 3G12 and 2D1 with the indicated synthetic peptide with sequences detailed in Table 1. All ELISA experiments were replicated with *N* = 4 and error bars show standard error of the mean

Using ELISA and different synthetic phosphorylated peptides (Table 2), the epitope specificity of antibody 3G12 was compared to other phospho-specific antibodies with nearby or overlapping epitopes (Fig. 4). As expected, antibody AT8 reacted with the synthetic pSer199/pS202/pSer205 peptide and not with any of the singly phosphorylated peptides (Fig. 4b). Based on previous studies, the AT8 antibody binding likely requires double phosphorylation preferentially at Ser202 and Thr205 [28]. Antibody CP13 is widely used and reported to be specific for tau phosphorylated at Ser202 [82], but limited published data is available about its properties. We confirmed that CP13 reacted with the synthetic peptide with pSer202, but it did not bind single pSer199, pThr205 or pSer208 peptides (Fig. 4c). However, CP13 also equivalently reacted with the pS199/pS202/pT205 peptide (Fig. 4c) showing that phosphorylation of these 2 amino acids in proximity of Ser202 did not influence its binding. 7F2 was reported as a monoclonal antibody that required phosphorylation of Thr205 for binding [73]; 7F2 bound to only the pThr205 peptide and not the pSer199, pSer202 or pSer208 peptide, but it can also bind when pSer199 and pSer202 are also phosphorylated (Fig. 4d). These additional studies further showed that antibody 3G12 only required tau peptide phosphorylated at Ser208 and not Ser199, Ser202 or Thr205 (Fig. 4e).

We previously made antibody 2D1 against the pSer199/pSer202/pThr205 peptide, but it was not phosphorylation specific [73]. We confirmed that 2D1 bound to this peptide sequence regardless of the specific phosphorylation state, but it prefers mono-phosphorylated peptide over the triple phosphorylated peptide (Fig. 4f). In addition, 2D1 did not bind to pSer208 or unphosphorylated Ser208 peptide, which begins at amino acid 202 indicating that the epitope of 2D1 antibody likely includes the 193–201 amino acid residues.

### Tau pSer208 is present in tau inclusions of different mouse models of tauopathies

**Fig. 5:**
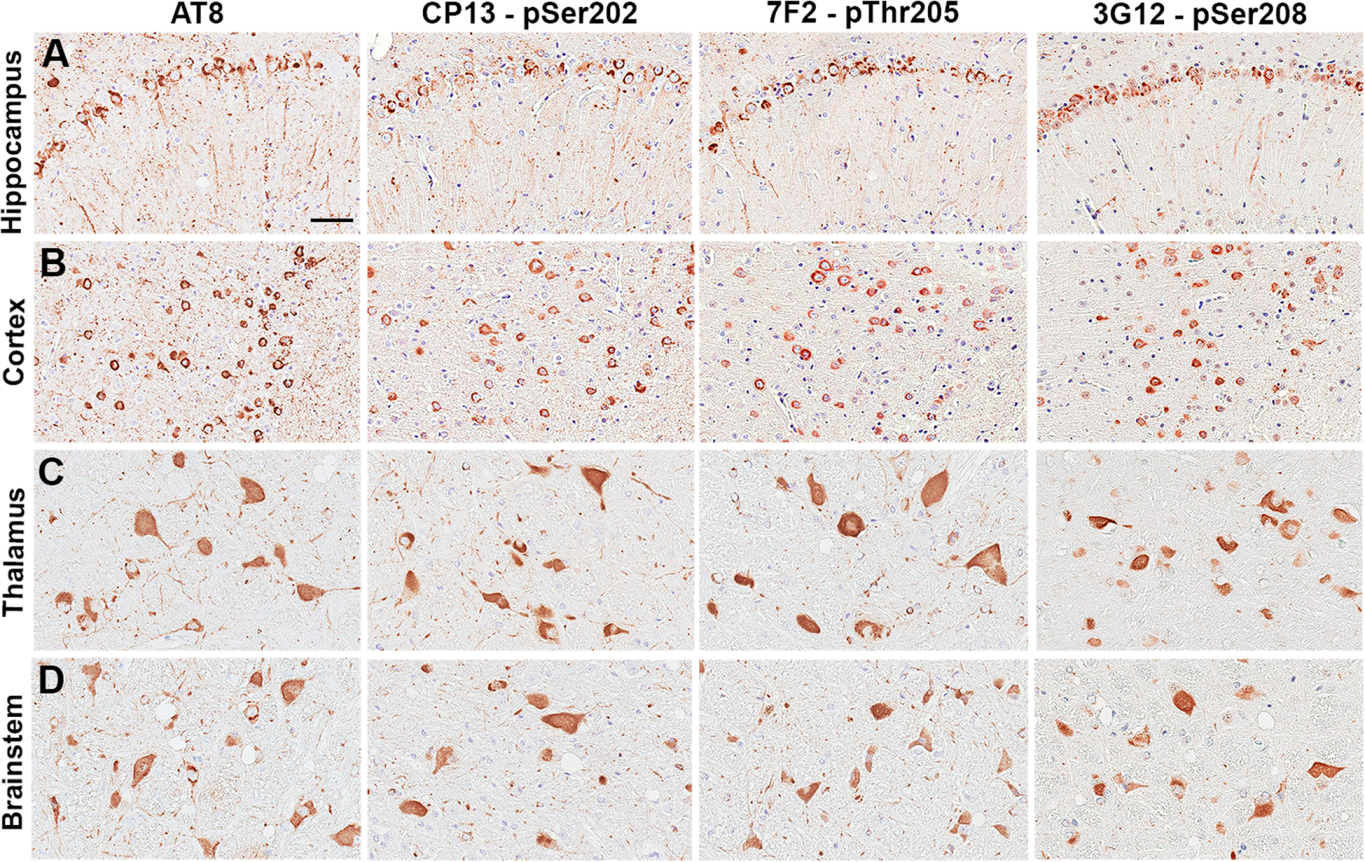
Tau phosphorylated at Ser208 is present in pathological inclusions of PS19 and rTg4510 transgenic mouse models of tauopathies. Staining of pSer208 specific antibody 3G12 is abundant in tau inclusions within the (**a**) hippocampus and (**b**) cortex of 6 month old rTg4510 tau transgenic mice compared to other phospho-tau specific antibodies AT8, CP13, and 7F2. Staining of pSer208 specific antibody 3G12 is abundant in tau inclusions within the (**c**) thalamus and (**d**) brainstem/pons of 12 month old PS19 tau transgenic mice compared to other phospho-tau specific antibodies AT8, CP13, and 7F2. Scale bar shows 50 μm

In the brains of two transgenic tau mouse models, 3G12 immunoreactivity was compared to other antibodies (AT8, CP13 and 7F2) against adjacent or overlapping phospho-epitopes. Adjacent brain sections from two mouse models were stained: rTg4510 transgenic mice that overexpress 0N4R human tau isoform with the P301L mutation specifically in the forebrain [67, 69] and PS19 transgenic mice that globally overexpress 1N4R human tau isoform with the P301S mutation in CNS neurons [85]. In aged 6-month-old rTg4510 mice, antibody 3G12 detected abundant tau inclusions in the cortex and hippocampus similar to antibodies AT8, CP13 and 7F2 on adjacent sections (Fig. 5a, b). This suggests that the tau inclusions in this model contain abundant pSer208 in a similar distribution. In the brains of aged PS19 mice, 3G12 antibody also detected tau inclusions in the thalamus and brainstem similarly to phosphorylation-specific antibodies AT8, CP13, and 7F2 (Fig. 5c, d).

### Tau Ser208 phosphorylation is a marker of mature NFT in AD patients

**Fig. 6:**
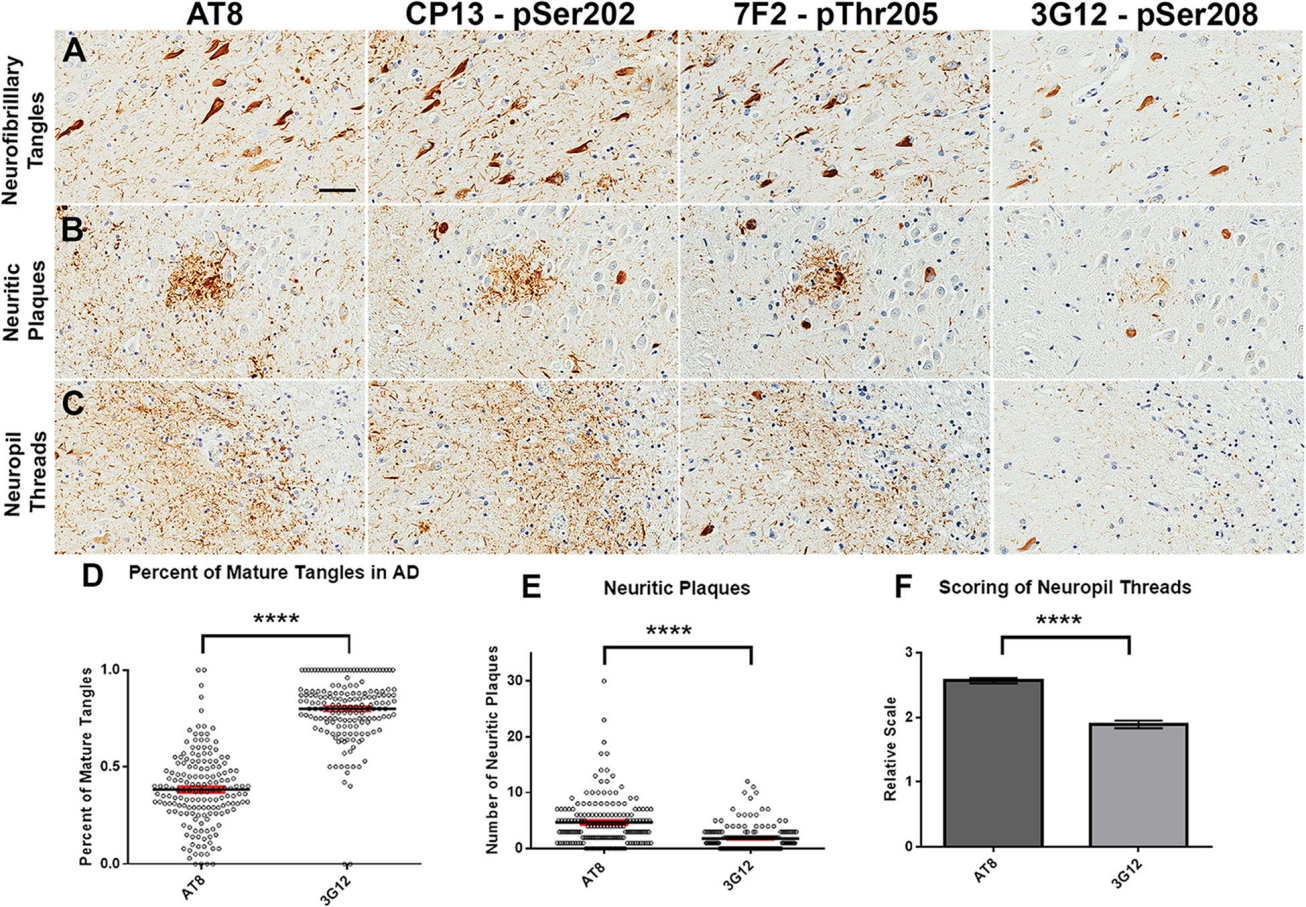
3G12 anti-tau pSer208 immunoreactivity for tau AD pathological inclusions compared to other phospho-tau specific antibodies with adjacent epitopes. Representative images of (**a**) NFT, (**b**) neuritic plaques, and (**c**) neuropil threads in the hippocampal formation of an AD patient with monoclonal antibodies AT8, CP13, 7F2 and 3G12. Scale bars represent 50 μm. (**d**) Quantification of percent of mature tangles over tau positive neurons stained by AT8 and 3G12 antibodies. Clear circles show individual data points. Error bars show 95% confidence intervals as represented by red bars. **e** Quantification of neuritic plaques stained by AT8 and 3G12. **f** Scoring of neuropil threads between AT8 and 3G12 staining

Next, we assessed if pSer208 is found in tau inclusions of post-mortem human brains from patients with different tauopathies. Brain tissue sections containing pathological tau inclusions from a series of patients with either AD or dementia with Lewy Bodies (DLB) with AD-tau pathology (summary of patients is shown in Table 3) were used. Hippocampal sections of these cases were stained with antibodies AT8, CP13, 7F2 and 3G12. AT8, CP13, and 7F2 showed similar staining for NFT, dystrophic neurites around neuritic plaques, and neuropil threads (Fig. 6). This is likely because these antibodies detect either pSer202, pThr205, or a combination of these 2 sites. 3G12 antibody against pSer208 showed preference for mature NFT as opposed to pre-tangles. 3G12 also detected less dystrophic neurites around neuritic plaques and fewer neuropil threads, suggesting that pSer208 may be a marker of late-stage aggregation specifically in NFT.

To further validate the qualitative differences between 3G12 and AT8 staining, we performed semi-quantitative counting of neuropil threads, neuritic plaques, and NFT in different microscopic fields across the hippocampal formation as described in “Materials and Methods.” The 3G12 antibody preferentially labeled mature NFT over less structured tau aggregates compared to AT8 (Fig. 6d). Similarly, 3G12 antibody demonstrated less reactivity both neuritic plaques and neuropil threads (Fig. 6e, f).

### In PSP and CBD brain samples, pSer208 immunoreactivity is selective for neuronal and oligodendroglial pathology over astrocytic pathology

**Fig. 7:**
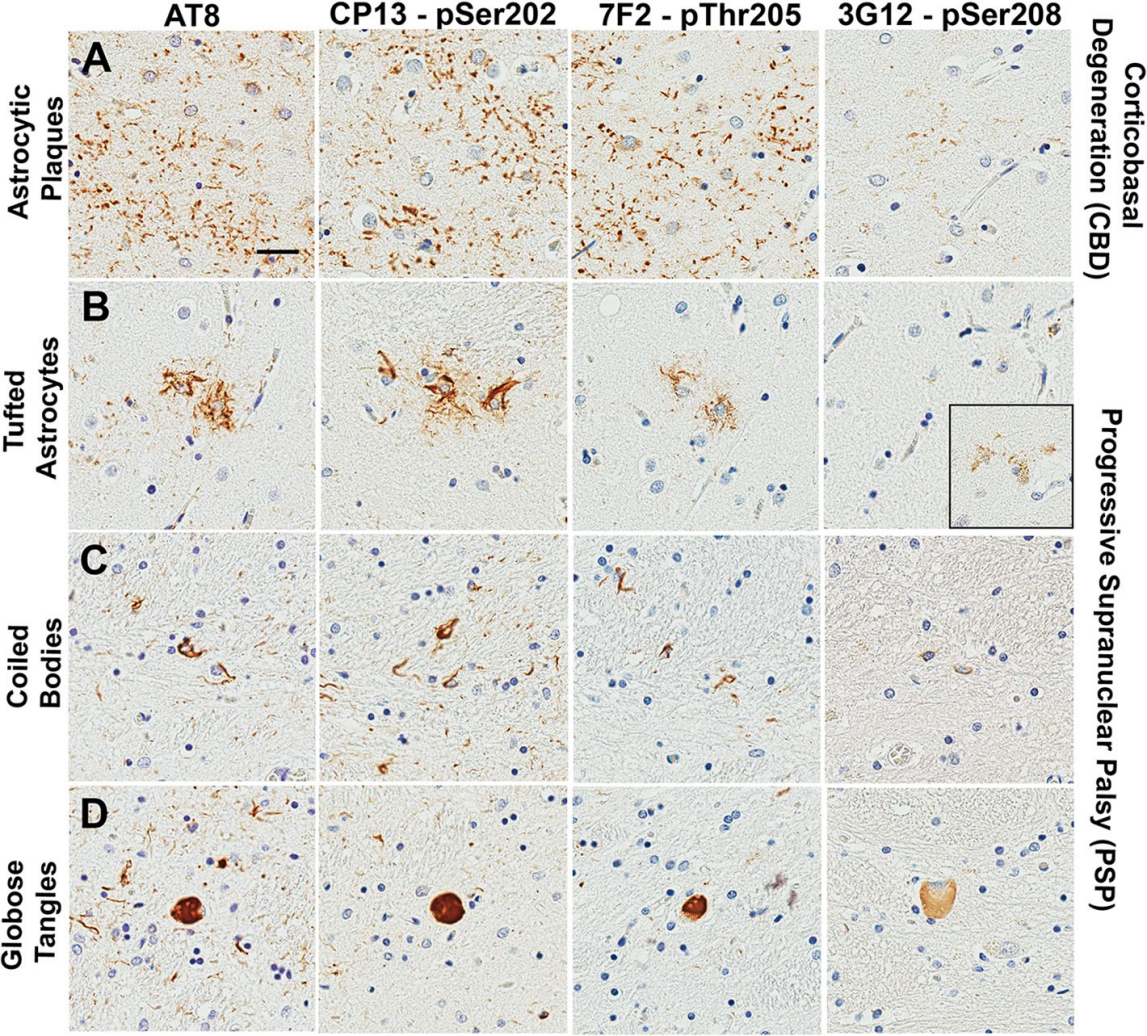
3G12 anti-tau pSer208 immunoreactivity for pathological inclusions in patients with PSP and CBD compared to other phospho-tau specific antibodies with adjacent epitopes. **a** Staining of astrocytic plaques in the striatum of CBD patients with monoclonal antibodies AT8, CP13, 7F2 and 3G12. Staining of (**b**) tufted astrocytes, (**c**) coiled bodies, and (**d**) globose tangles in the striatum of PSP patients. Scale bar shows 25 μm

In addition to AD and DLB cases, tau phosphorylation patterns were investigated for other tauopathies such as PSP and CBD. In the brain tissue of patients with CBD, AT8, CP13, and 7F2 antibodies stained astrocytic plaques in a similar manner (Fig. 7a). The 3G12 antibody showed significantly less staining for astrocytic plaques and many of these plaques were not captured. In PSP brains, a similar selectiveness was observed where 3G12 revealed limited staining of tufted astrocytes (Fig. 7b). In fact, barely any tufted astrocytes were detected with 3G12, while AT8, CP13, and 7F2 abundantly stained tufted astrocytes. Interestingly, 3G12 antibody stained neuronal globose tangles and oligodendroglial coiled bodies with the same distribution as AT8, CP13, and 7F2 antibodies (Fig. 7c, d). Although staining intensity was less, 3G12 antibody appeared to capture most of the neuronal and oligodendroglial pathology labeled by the other antibodies. These data suggest that pSer208 in PSP is more prominent in neuronal and oligodendroglial inclusions than in astrocytic inclusions.

## Discussion

Elevated tau phosphorylation at over 45 sites is a hallmark of AD brain PHF-tau and many of these post-translational modifications occur within the proline-rich region of tau including Ser202, Thr205 and Ser208 [34]. Phosphorylation-specific antibodies such as AT8 can react with multiple combinations of pSer202, pThr205, and pSer208 [53, 65] and these antibodies are important for postmortem Braak staging of AD [11]. Nevertheless, tau phosphorylation at Ser208 remains poorly studied, although recent in vitro studies have indicated that Ser202/Thr205/Ser208 combined phosphorylation can promote tau aggregation without the addition of typical chemical inducers [19].

Due to difficulties with studying single phosphorylation specificity by kinases in vivo, phosphomimetic alterations were used to model site-specific phosphorylation of Ser202, Thr205, and Ser208 in cell culture. Specific Ser or Thr residues were mutated to Glu that act to mimic pSer or pThr respectively, in terms of size and charge. Previous studies have shown that tau phosphomimetics can adopt pro-aggregative conformations like in AD [7, 41]. From our phosphomimetic experiments, we found that triple phosphomimetic S202E/T205E/S208E promoted tau aggregation in cell culture, but phosphomimetic of combined S202E/T205E and only S208E did not by themselves (Fig. 1). This finding indicated that Ser208 phosphorylation can be an enhancer of tau aggregation when Ser202/Thr205 are also phosphorylated. These results are congruent with previous in vitro experiments [19].

The studies were extended to assess the role of prion-like seeding on tau aggregation by adding recombinant K18 tau seeds to different phosphomimetics, since it has been suggested that tau may propagate in a prion-like mechanism [5, 27]. None of the phosphomimetics including triple phosphomimetic S202E/T205E/208E were modulated by K18 seeding. Since P301L can be enhanced by K18 seeding as previously demonstrated (Fig. 1c) [72, 84], we added the triple phosphomimetic S202E/T205/S208E to P301L. Surprisingly, there was not an additive or enhanced aggregation in this quadruple mutation of S202E/T205E/S208E/P301L. Based on these results, P301L may represent a different tau aggregation species that is distinctively enhanced by seeding and operates in a different mechanism separate from this phosphorylation change. In our prior screenings of more than 31 different tau mutants [72, 84], we demonstrated that P301L and related mutations in the P301 position are uniquely prone to aggregation induced by K18 tau seeds. Therefore, our findings indicate that two types of modifications that individually enhance aggregation are not necessarily additive if the molecular alterations and conformations are not compatible to promote further misfolding.

A major function of tau is to bind and stabilize MTs [42, 83], which can be regulated by the phosphorylation of amino acid residues like serine, threonine, or tyrosine [22, 47, 49, 71]. Using a cell-based assay [84], the effects on MT binding of different phosphomimetics studies were assessed. S208E was found to display increased MT binding, which acts similar to disease-causing tau mutants R5H and R5L [84]. Abnormal MT binding may impair the ability of tau to properly regulate MT assembly and dynamics. Surprisingly, the double phosphomimetic S202E/T205E and triple phosphomimetic S202E/T205E/S208E did not significantly alter MT binding. This may be a limitation of using multiple phosphomimetics to model MT function, because it has been previously demonstrated that the effects are not necessarily additive [26, 47], likely due to complex changes in protein structure/folding.

A monoclonal antibody (3G12) specific to tau phosphorylated at Ser208 was generated to allow to monitor this modification in animal models and human brain tissue. The specificity of the 3G12 antibody was compared to the AT8 antibody and other related antibodies against nearby sites. Although AT8 antibody binds around the center of pSer202/pThr205 [24, 28], past studies have shown that AT8 can also react with pS199 and pSer208 but is highly selective for dual amino acid phosphorylation within its binding site [53, 65] consistent with the data here. This specificity of AT8 is likely because it was created by immunizing mice with purified PHF-tau from AD brains which is phosphorylated at many of these residues within the binding interaction of AT8 [58]. CP13 is a widely used tau monoclonal antibody with reported specificity for tau phosphorylated at pSer202 [82], but little experimental data is actually available on the characterization of this epitope. Using synthetic peptides, it is confirmed that CP13 recognizes tau phosphorylated at Ser202 and not Ser199 or Thr205, but in addition phosphorylation at both Ser199 and Thr205 do not interfere with CP13 binding which is also probably because this antibody was also raised against PHF-tau from AD brains [82].

In two different mouse models of tauopathies, our 3G12 antibody robustly stained tau inclusions in cortex and hippocampus of rTg4510 mice, and in thalamus and brainstem of PS19 mice. These two mouse models expressed different tau mutations and variants: PS19 mice overexpress the 1N4R human isoform with the P301S mutation [85] and rTg4510 transgenic mice overexpressed the 0N4R human isoform with the P301L mutation [67, 69]. These findings indicate that Ser208 is phosphorylated within the pathological inclusion that accumulated in these different animal models similarly to AT8, CP13, and 7F2 antibody staining, which correspond to phosphorylation at Ser202 and Thr205.

In AD and DLB patients with AD-tau pathology, it is shown that 3G12 antibody reveals strong preference for mature tangles relative to pre-tangles and other unstructured aggregates of tau compared to AT8. This observation was consistent across multiple regions of the medial temporal lobe. pSer208 is unique as a marker of aggregation and mature NFT compared to the other phosphorylation sites that stain all forms of tau. Phosphorylation at Ser208 might occur sequentially after Ser202 and Thr205 phosphorylation as the S208E phosphomimetic significantly reduces phosphorylation of these sites as determined by phosphorylation-specific antibodies AT8, CP13, and 7F2, at least in HEK293T cells (Supplemental Figure 2). Since Braak staging is based on the distribution of mature NFT [10, 11], pSer208 will be useful for both early and late stage AD to track all Braak stages I to VI. An aggregation-specific marker like pSer208 could make diagnosis easier, since it does not pick up extraneous staining of uncertain significance. The other antibodies CP13 and 7F2 revealed similar staining patterns as AT8, but might be more specific for pathological tau aggregates, as AT8 has been shown to cross-react with MAP 2C [54, 73].

To further investigate phosphorylation differences in other tauopathies, PSP and CBD cases were also selected for histopathologic analysis (Table 3). 3G12 immunoreactivity showed a distinct preference for neuronal and oligodendroglial pathology in PSP and detected less astrocytic pathology in either CBD or PSP. There were no major differences in AT8, CP13, or 7F2 staining in PSP and CBD, which suggests that Ser208 may be phosphorylated at different levels in different types of PSP and CBD pathology. It is not entirely surprising that different types of tau pathology can have variations in phosphorylation patterns; however, many phosphorylation-specific antibodies like AT8 and PHF-1 do not stain PSP and CBD pathology differently [21]. Recent cryo-EM studies suggest that CBD tau filaments may have different structures compared to AD tau filaments [86]. PSP tau filaments likely also adopt a different structural conformation [4]. Experimentally, PSP and CBD tau pathology can be propagated as distinctively different strains when injected into the brains of different transgenic tau mouse models [14, 61, 62]. Since post-translational modifications can differentiate CBD from AD [3], it is possible that CBD or PSP pathology represent different tau conformations with distinctively different phosphorylation patterns that define their structure and properties.

**Fig. 8:**
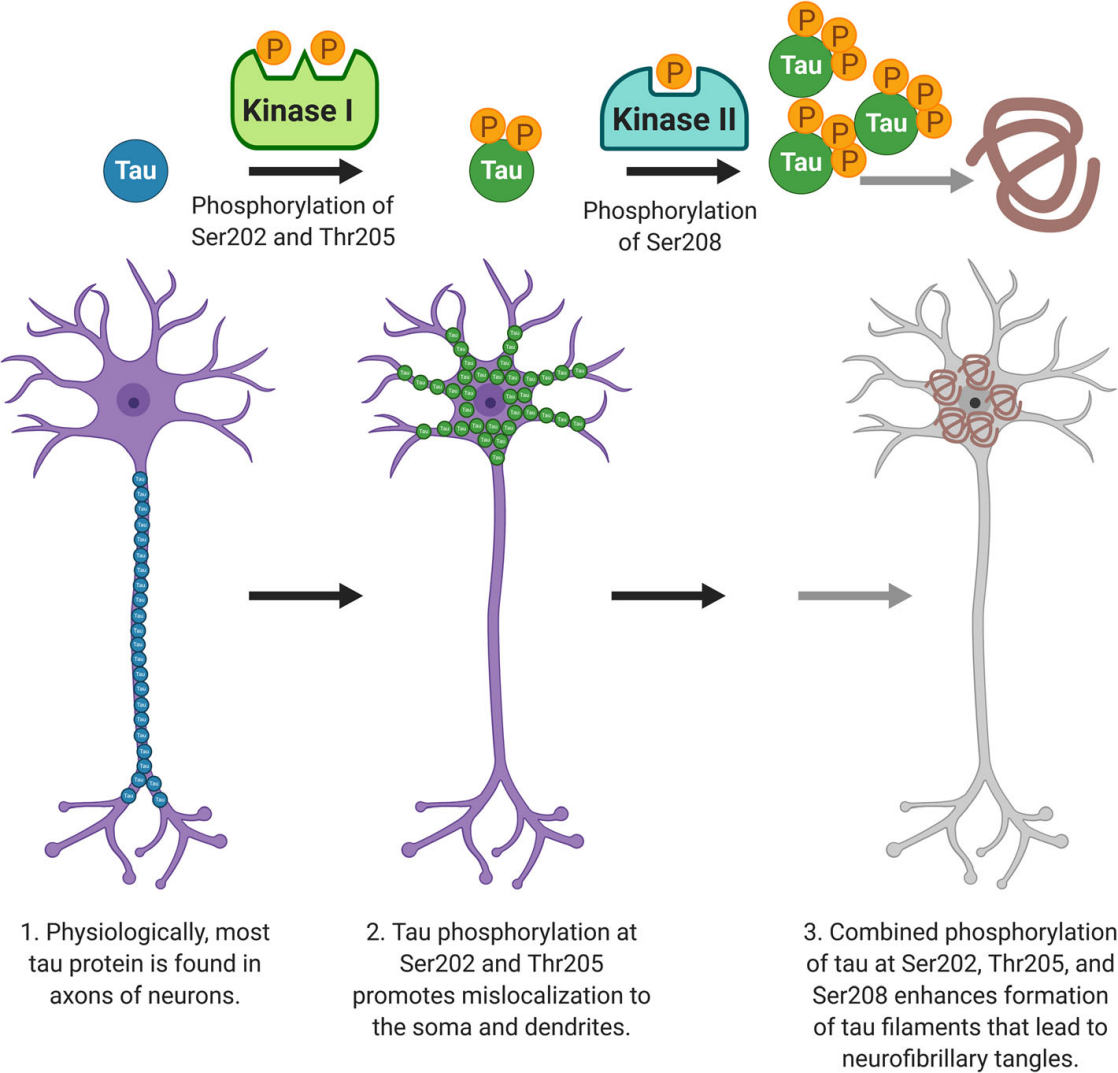
Triple phosphorylation of Ser202, Thr205, and Ser208 promotes tau mislocalization and aggregation, leading to the formation of NFT. 1) Physiologically, most tau protein is found in neuronal axons. 2) Tau phosphorylation at Ser202 and Thr205 leads to tau mislocalization to the soma and dendrites. 3) Combined phosphorylation of tau at Ser202, Thr205, and Ser208 enhances formation of tau filaments that lead to neurofibrillary tangles. Figure was created with Biorender

Based on our results and previous studies, we propose a model for how tau hyperphosphorylation near the AT8 epitope might lead to tau aggregation (Fig. 8). Normally, most tau protein is predominantly found in axons and associated with MTs. Phosphorylation of Ser202 and Thr205 may promote tau to mislocalize from the axon to the soma and dendrites, often detected as pre-tangles by the AT8 antibody [10, 11]. Combined phosphorylation of Ser202, Thr205, and Ser208 forms a unique post-translational modification configuration that promotes tau aggregation, accelerating the formation of tau filaments, and eventually resulting in NFT.

Phosphorylation of Ser202 and Thr205 is likely regulated by different types of kinases compared to phosphorylation of Ser208. Ser202 and Thr205 have been shown to be phosphorylated by proline-directed kinases such as mitogen-activated protein kinases (MAPK) [68], cyclin-dependent kinase 5 (CDK5) [46] and glycogen stimulated kinase-3β (GSK-3β) when primed with protein kinase A (PKA) [50, 51]. In contrast, non-proline directed kinases like checkpoint kinase 1 (Chk1) [57], casein kinase 1 delta (CK-1) [36] and tau-tubulin kinases (TTK) [76] have been shown to phosphorylate Ser208, but none of these kinases reportedly phosphorylate Ser202 and Thr205. This suggests that two distinct groups of kinases phosphorylate Ser202 and Thr205 compared to Ser208. Phosphorylation of Ser208 likely occurs at a different disease stage from phosphorylation of Ser202 and Thr205. It is also possible that different tauopathies may have variations in phosphorylation patterns that define different tau conformations and species.

Besides histopathological diagnosis, an antibody specific for mature tangles such as 3G12 might provide high potential diagnostic value as a blood and cerebrospinal fluid (CSF) biomarker for NFT progression in AD patients, as pSer208 was reported to be distinctively elevated in the CSF of AD patients compared to healthy controls [6]. Antibody 3G12 could also be used as immunotherapy for specific targeting of mature tangles in AD as several phosphorylation-specific antibodies have shown efficacy in different tau mouse models and in developing clinical trials [15, 45, 56, 79].

## Supplementary information

**Additional file 1: Figure S1.** Soluble fractions of WT tau, tau mutant P301L, and tau phosphomimetics have similar expression levels. Soluble fractions of WT tau, P301L, and phosphomimetics S208E, S202E/T205E, S202E/T205E/S208E, S202E/T205E/S208E/ P301L were immunoblotted with a total tau antibody 3026. The relative molecular masses of protein markers are indicated on the left.

**Additional file 2: Figure S2.** Tau S208E phosphomimetic presents significantly reduced phosphorylation of nearby sites Ser202 and Thr205. HEK293T cells were transfected to express WT tau or S208E tau phosphomimetic 2N4R isoform. Whole cell lysates were immunoblotted with a total tau antibody 3026 and phosphorylation specific antibodies CP13, 7F2, and AT8. The relative molecular masses of protein markers are indicated on the left.

## Data Availability

All data generated or analyzed during this study are included in this published article.

## Abbreviations

AD: Alzheimer’s disease
ADNC: Alzheimer’s disease with neuropathologic change
Aβ: Beta-amyloid
ABC: Avidin-biotin complex
ARTAG: Aging-related tau astrogliopathy
CAA: Cerebral amyloid angiopathy
CBD: Corticobasal degeneration
CSF: Cerebrospinal fluid
CTRND: Center for Translational Research in Neurodegenerative Disease
DAB: 3,3′-diaminobenzidine
DLB: Dementia with Lewy bodies
ELISA: enzyme-linked immunosorbent assay
FBS: Fetal bovine serum
FTLD-tau: Frontotemporal lobar dementia-tau
HEK293T: Human embryonic kidney 293 cell line with SV40 large T antigen
KO: Knockout
LATE: Limbic-predominant age-related TDP-43 encephalopathy
MAPT: Microtubule-associated protein tau
MT: Microtubules
NFT: Neurofibrillary tangles
nTG: Nontransgenic
PBS: Phosphate buffered saline
PHF: Paired helical filaments
PSP: Progressive supranuclear palsy
TMB: 3,3′,5,5′-tetramethylbenzidine
UF HBTB: University of Florida Neuromedicine Human Brain and Tissue Bank
WT: Wild type
