# Supplementary figures and images for: Tau Ser208 phosphorylation promotes aggregation and reveals neuropathologic diversity in Alzheimer’s disease and other tauopathies
Source: Acta Neuropathol Commun. 2020 Jun 22;8:88. doi: 10.1186/s40478-020-00967-w (PMC7310041; doi:10.1186/s40478-020-00967-w)

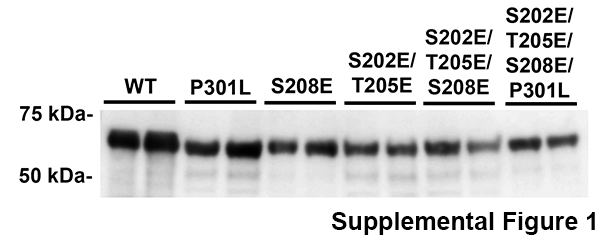

Supplement: Supplementary file 1 — Additional file 1: Figure S1. Soluble fractions of WT tau, tau mutant P301L, and tau phosphomimetics have similar expression levels. Soluble fractions of WT tau, P301L, and phosphomimetics S208E, S202E/T205E, S202E/T205E/S208E, S202E/T205E/S208E/ P301L were immunoblotted with a total tau antibody 3026. The relative molecular masses of protein markers are indicated on the left. [file 40478_2020_967_MOESM1_ESM.tif]

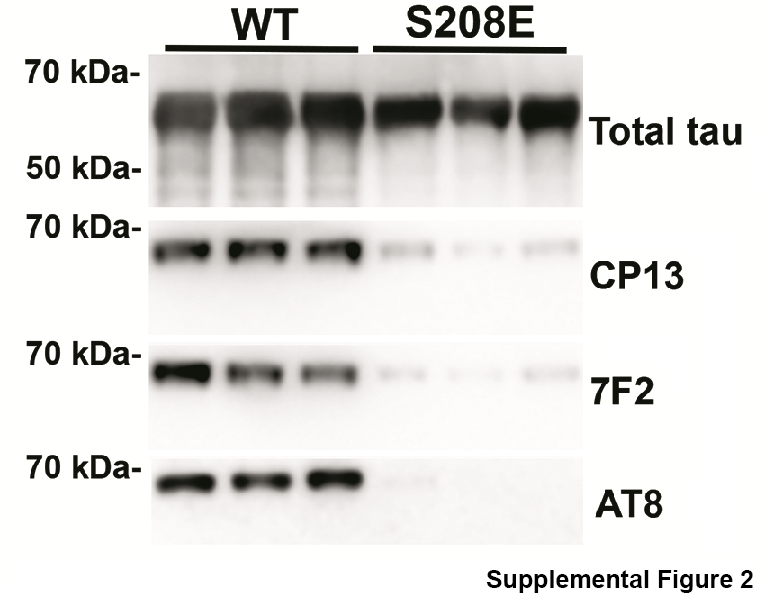

Supplement: Supplementary file 2 — Additional file 2: Figure S2. Tau S208E phosphomimetic presents significantly reduced phosphorylation of nearby sites Ser202 and Thr205. HEK293T cells were transfected to express WT tau or S208E tau phosphomimetic 2N4R isoform. Whole cell lysates were immunoblotted with a total tau antibody 3026 and phosphorylation specific antibodies CP13, 7F2, and AT8. The relative molecular masses of protein markers are indicated on the left. [file 40478_2020_967_MOESM2_ESM.tif]
